# Whole exome sequencing identified a novel truncation mutation in the *NHS* gene associated with Nance-Horan syndrome

**DOI:** 10.1186/s12881-018-0725-3

**Published:** 2019-01-14

**Authors:** Chao Ling, Ruifang Sui, Fengxia Yao, Zhihong Wu, Xue Zhang, Shuyang Zhang

**Affiliations:** 1 Laboratory of Clinical Genetics, Peking Union Medical College Hospital, Chinese Academy of Medical Sciences and Peking Union Medical College, Beijing, 100730 China; 2 Department of Ophthalmology, Peking Union Medical College Hospital, Chinese Academy of Medical Sciences and Peking Union Medical College, Beijing, 100730 China; 3 Central Research Laboratory, Peking Union Medical College Hospital, Chinese Academy of Medical Sciences and Peking Union Medical College, Beijing, 100730 China; 4 McKusick-Zhang Center for Genetic Medicine, State Key Laboratory of Medical Molecular Biology, Institute of Basic Medical Sciences, Chinese Academy of Medical Sciences, School of Basic Medicine, Peking Union Medical College, Beijing, 100005 China; 5 Department of Cardiology, Peking Union Medical College Hospital, Chinese Academy of Medical Sciences and Peking Union Medical College, Beijing, 100730 China

**Keywords:** Congenital cataract, Nance-Horan syndrome, Hereditary, *NHS* mutation

## Abstract

**Background:**

Nance-Horan syndrome (NHS) is an X-linked inheritance disorder characterized by bilateral congenital cataracts, and facial and dental dysmorphism. This disorder is caused by mutations in the *NHS* gene. However, NHS may be difficult to detect in individuals with subtle facial dysmorphism and dental abnormalities in whom congenital cataracts are the primary clinical manifestations.

**Methods:**

In this study, we present a three-generation family with NHS. Whole exome sequencing was performed to determine the potential pathogenic variant in the proband. Further validation was explored with Sanger sequencing in 9 of the available individuals of the family and additional 200 controls.

**Results:**

A novel truncation mutation in gene *NHS* (c.C4449G, p.Tyr1483Ter) was found in the proband, who presented with a long-narrow face, prominent nose and large anteverted pinnae ear, screw-driver like incisors, mild mulberry like molars, one missing maxillary second molar and malocclusion. We found this mutation was detected in 2 male patients and 4 female carriers in the family. However, the mutation was never detected in the control subjects.

**Conclusions:**

In conclusion, we identified a novel truncation mutation in the *NHS* gene, which might associate with NHS. Our review on the NHS studies illustrated that NHS has significantly clinical heterogeneity*.* And *NHS* mutations in the NHS-affected individuals typically result in premature truncation of the protein. And the new mutation revealed in this study would highlight the understanding of the causative mutations of NHS.

**Electronic supplementary material:**

The online version of this article (10.1186/s12881-018-0725-3) contains supplementary material, which is available to authorized users.

## Background

Nance-Horan syndrome (NHS) is a rare X-linked genetic disorder. According to the latest statistics of the National Organization for Rare Disorders shown that fewer than 50 families have been described in the medical literatures, and the exact incidence of the disorder remains unknown (https://rarediseases.org/rare-diseases/nance-horan-syndrome/). NHS is caused by a mutation in the *NHS* gene on chromosome Xp22. NHS is usually fully expressed in males only, with the affected males characterized by congenital cataracts and frequent microcornea, dental anomalies, and dysmorphic features [1, 2]. Approximately 20 to 30% of affected males may have varying levels of mental retardation [3]. Autism spectrum disorder symptoms have also been observed in a few patients [4]. Carrier females may show posterior Y-sutural cataracts with small corneas and slightly reduced vision only, they do not usually develop intellectual impairment [5, 6]. Since the symptoms of isolated microphthalmia with cataract; cataract-microcornea syndrome; X-linked congenital cataract and X-linked congenital cataract with microcornea are similar to those of NHS, making a definitive diagnosis without genetic information is difficult. Thus, to determine the potential disease-causing gene, we performed whole exome sequencing (WES) analysis in a proband.

## Methods

### Mutation screening

Peripheral blood was collected in potassium EDTA tubes from the three generations of family members. Genomic DNA was extracted with a QIAamp DNA blood kit (Qiagen, CA, USA) according to the manufacturer’s recommendations. Subsequent DNA quantification was performed with a NanoDrop ND-2000 spectrophotometer (https://www.labtech.com/nanodrop-nd2000-nd2000c-spectrophotometer) and analyzed with agarose gel electrophoresis. WES was explored with NimbleGen VCRome 2.1 capture reagents and sequenced by an Illumina HiSeq platform. The produced sequencing reads were aligned to the reference human genome (GRCh37/hg19) using the Burrows-Wheeler algorithm (version 0.7.5). Exome variants were identified with GATK workflows and annotated with the ANNOVAR pipeline.

### In silico analyses and sanger sequencing

Variants were filtered for relevance to human disease based on a minor allele frequency greater than 0.01 in the 1000 Genomes, ExAC and ESP6500 databases: missense, nonsense, indel, and splice-site variants were identified. These candidate variants were further analyzed with SIFT, Polyphen 2, CADD and Mutation Taster (http://mutationtaster.org/) software to predict the probability of disease-causing mutations. Variant interpretation was explored with the InterVar pipeline [7]. The American College of Medical Genetics and Genomics (ACMG) standard was used as the final criterion to evaluate the pathogenicity of the variants. Sanger sequencing was performed to validate the variant sites in the proband as well as in 9 available family members using designed primers (forward primer 5’-TCAGACTGTGGGTGCTACAAGG-3′ and reverse primer 5’-AGCCTTCTGCATTAACTCGTGG-3′). In addition, candidate disease-causing mutations were examined in 200 control subjects, and all of the sequence traces were analyzed with Mutation Surveyor software [8].

## Results

### Patients and clinical characteristics

**Fig. 1:**
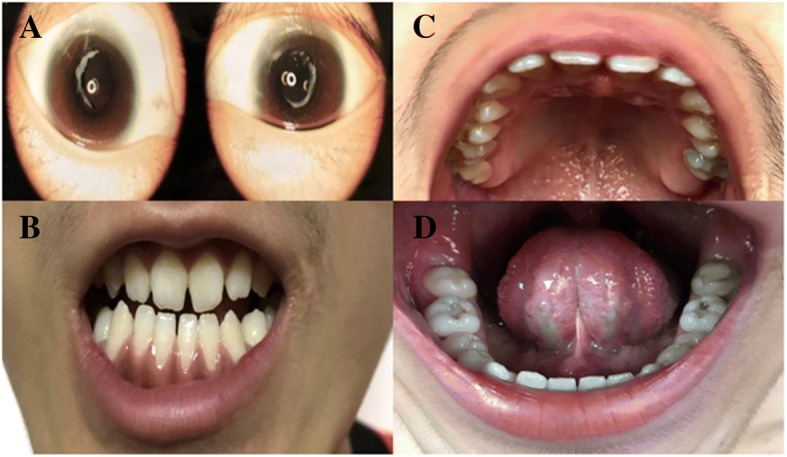
Main clinical manifestations of the proband. **a** Slit lamp photograph showing that the proband presented with bilateral congenital cataracts; **b** The screwdriver blade-shaped incisors; **c** One missing maxillary second molar; **d** The mild mulberry-like molars

A 15-year-old boy was admitted to the laboratory of clinical genetics of Peking Union Medical College Hospital for genetic counseling due to congenital cataracts. In addition to the primary physical characteristic of congenital bilateral nuclear cataracts, the young patient had a long-narrow face, prominent nose and large anteverted pinnae, screw-driver like incisors, mild mulberry like molars, one missing maxillary second molar and malocclusion (Fig. 1). Available clinical information regarding the families is shown in Table 1, and the carrier females lens opacity and vision were unavailable here.

**Table 1:** Manifestations of the available family members

ID	Gender	Age	Cataract	Nystagmus	Dental anomalies
I-1	Male	60	N	N	N
I-2	Female	60	N	N	N
II-1	Female	36	N	N	N
II-2	Female	34	N	N	N
II-3	Female	32	N	N	N
II-4	Female	30	N	N	N
II-5	Male	28	Y	Y	Y
II-6	Male	26	N	N	N
III-1 (Proband)	Male	15	Y	N	Y

*Y* Yes, *N* No

**Fig. 2:**
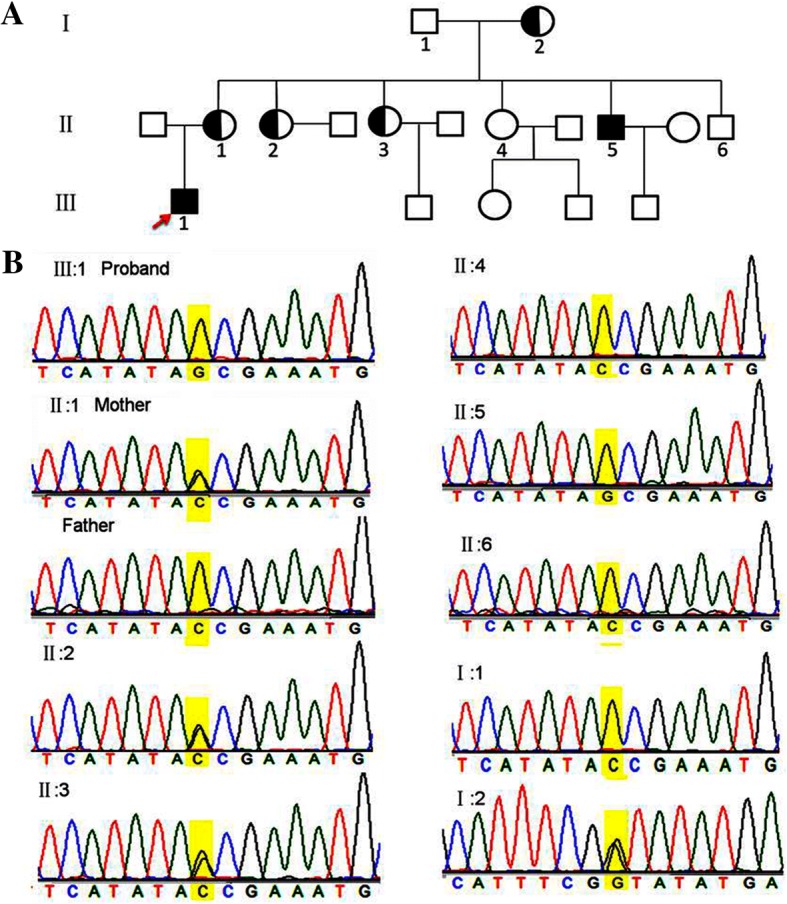
The pedigree and Sanger identification of the novel mutation. **a** The black-solid square represents the affected male patient. The black-shadow circle represents the female carrier. Unfilled squares and circles indicate normal males and females respectively. **b** The Sanger sequencing traces illustrated the *NHS* c.C4449G, p.Tyr1483Ter mutation in individualsI:2, II:2 and II:3 (hemizygous), and individuals II:5 and III:1 (homozygous)

The proband (III:1) and another affected individual (II:5) underwent microincision cataract surgery at the ages of 15 and 21 years respectively. Available photos before the surgery revealed bilateral lens opacities that heavily affected the nucleus in the proband (Fig. 1; Fig. 2). Individual II: 5 presented with similar facial features including a long narrow face, mildly anteverted pinnae ear and screw-driver like incisors, mild mulberry like molars and malocclusion (Additional file 1). Individual II: 5 stated that he was affected by nystagmus, and at the time of recruitment in our study, which might associate with NHS. However, neither of these individuals had mental retardation or behavioral disturbances. Their family history revealed no obvious visual defects or dental abnormalities among the rest of the family members.

### A novel mutation associated with NHS

**Fig. 3:**
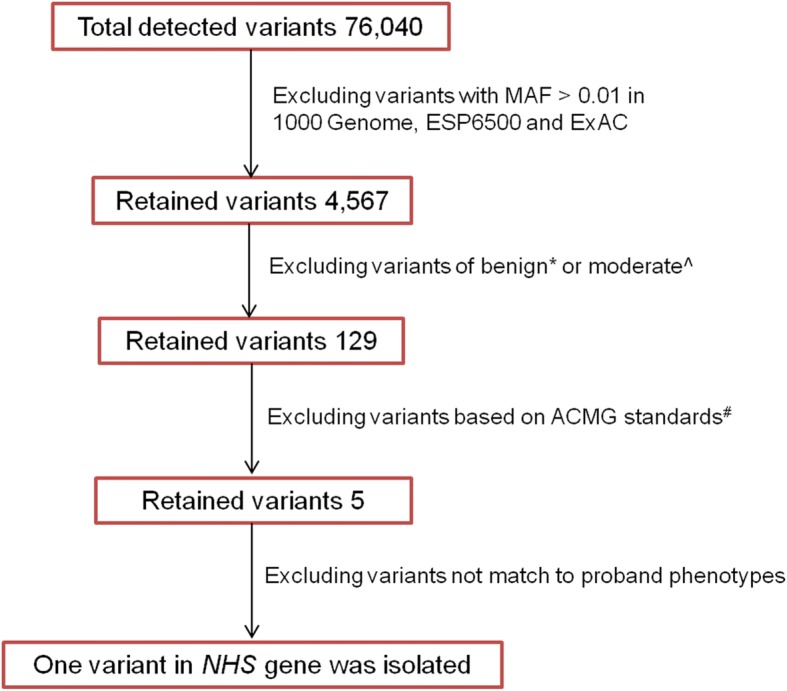
Variant filter flow chart. To identify the underlying variant, we performed strict filter procedures. Benign*: variants occurred in the noncoding region and no prediction indicated pathogenic or likely pathogenic. Morderate^: variants occurred in the coding region, but no prediction implied pathogenic or likely pathogenic ACMG standards: variants were classified with the rules of combining criteria of the ACMG guidelines, and 5 of the pathogenic variants were identified

WES data were processed as described in the Methods section. Quality control parameters included an average coverage of 100× of the exome, and 97.6% of exonic regions sequenced at greater than 10× coverage. A total number of 63.6 million reads were mapped to the reference genome. After annotation 76,040 variants were detected in the proband. Variant isolation was performed with a strict workflow (Fig. 3), and 129 coding variants were selected with high priority, According to ACMG standards, 5 genes were identified and predicted to be pathogenic with very strong evidence [7], however, only the *NHS* gene was associated with cataract and NHS (Additional file 2). Thus, based on the family pathogenic segregation study and clinical manifestations, a novel stop-gain mutation c.C4449G, p.Tyr1483Ter in *NHS* was identified. We further validated this mutation in 9 available family members; it was detected in individual II: 5 and 4 female carriers, but not in the 200 controls (Fig. 2 and Additional file 3). Here, we predicted that this novel mutation was most likely associated with NHS.

## Discussion

In this study, we revealed a novel truncation mutation (c.C4449G, p.Tyr1483Ter) in the *NHS* gene, which might associated with NHS in a three-generation Chinese family. The whole coding sequence of *NHS* gene is 4893 bp, and in our study the novel truncation occurred in c.4449 site, and the affected individuals in the family presented with relatively mild abnormality. Although the male affected individuals had the same variation, the phenotypic heterogeneity existed in the NHS family (Table 1). This implied that the underlying mechanism of clinical heterogeneity was still needed to be investigated.

NHS manifestations appear in multiple organ systems especially the features of facial, teeth, skeleton, heart and neurologic system features, and even encompass behavior. And heterozygous females commonly had posterior Y-sutural cataracts. In this study we had no chance to perform the professional ophthalmologic examination for the carrier females, since they do not think they have vision impairment, and their lens opacity and vision was not quite clear. However, X-linked cataract merely involves the eyes. Heterozygous females had posterior stellate or suture cataracts, or a combination of the two, with normal vision or a slight reduction (Table 2). However, the facial dysmorphology and dental abnormalities might be subtle and easily missed in NHS patients in whom congenital cataracts are the primary clinical concern. Additionally, there was not enough evidence to identify the two disorders that might be allelic [9].

**Table 2:** The manifestations of Nance-Horan syndrome and X-linked cataract

	Nance-Horan syndrome (John F. Jackson created on 6/15/1995 and Kelly A. Przylepa revised on 6/30/2004)	X-linked cataract
Inheritance	X-linked dominant	X-linked
Face	Long, narrow face	Normal
Ears	Large anteverted pinnae (90% males, 40% females)	Normal
Eyes	Bilateral congenital cataracts (males)	Congenital nuclear cataract in males
	Vision loss, profound (males)	Severe visual impairment in males
	Microcornea	Pronounced microcornea
	Nystagmus	Heterozygous females had posterior suture or posterior stellate cataracts, or a combination of the two, with normal or slight reduction in vision.
	Microphthalmia	
	Posterior Y-sutural cataracts (females)	
	Normal vision (females)	
	Glaucoma (~ 50% of males)	
Nose	Prominent nose and nasal bridge	Normal
Teeth	Screwdriver blade-shaped incisors (males and females)	Normal
	Supernumerary maxillary incisors (mesiodens) (~ 65% males)	
	Tapered premolar and molar cusps	
	Diastema (males and females)	
Skeletal	Broad fingers; short fingers	Normal
Heart	Congenital heart defects	Normal
Neurologic	Mild-moderate mental retardation (~ 80% affected males)	Normal
Behavioral Psychiatric Manifestations	Behavioral disturbances Autism	
Gene mutation	*NHS*	*NHS*

The *NHS* gene is alternatively spliced and composed of 10 coding exons, which encode a protein containing 4 conserved nuclear localization signals, The encoded protein can regulate actin remodeling and cell morphology. Expression studies in mice showed that the *NHS* gene plays critical roles in regulating eye, tooth, brain, and craniofacial development. Additionally, the complex pattern of temporally and spatially regulated expression of *NHS* was consistent with the pleiotropic features of NHS [3].

Previous studies illustrated that variations in the *NHS* gene could induce X-linked cataracts and NHS [10–12]. However, the identical association between the genotype and phenotype was unclear. One study predicted that a lack of functional *NHS* protein might cause NHS, whereas aberrant transcription of the *NHS* gene might lead to a milder X-linked cataract phenotype [10]. To determine whether NHS and X-linked cataract were the two distinguishing phenotypes of *NHS* gene mutations, we reviewed the pathogenic/likely pathogenic mutations, which were recorded in the ClinVar and Cat-Map databases [13]. Here, more than 50 mutations of the *NHS* gene accounted for pathogenic clinical conditions. Variants including missense, frame-shift (InDel) and stop-gain mutations were identified as contributors to NHS; and a 4.8 kb deletion and a 500 kb triplication were associated with X-linked cataract 40 (Additional file 4); Considering the genotypes and phenotypes of NHS and X-linked cataracts, we found that NHS was mostly resulted from the point mutation and micro InDel [14–16], while the X-linked cataract was caused by large-scale recombination of the *NHS* gene, which was predicted to result in altered transcriptional regulation of the *NHS* gene. However, this difference could not be used to define NHS and X-linked cataract [9], since a microdeletion of 170,6 kb at Xp22.13 was detected in an Italian boy with NHS syndrome [15]. The latest cytogenetic and molecular analyses in two patients, a mother and daughter, who presented with NHS, demonstrated a 46, X, t(X;1) (p22.13;q22) karyotype in each patient. No copy-number genomic imbalances were detected. The mother had a preferential inactivation of the normal X chromosome and no mRNA isoform of *NHS* was detected. This study implied that NHS could be due to the disruption of *NHS* gene expression [12].

NHS-affected individuals have mutations in the *NHS* gene that typically result in premature truncation of the protein (Additional file 4). The mutant proteins failed to localize to the cellular periphery in epithelial cells and instead were found in the cytoplasm. The mislocalization of the mutant NHS-A protein is expected to adversely affect cell-cell junctions in epithelial cells such as the lens epithelium, which may explain the cataractogenesis in NHS patients [10, 17, 18], and *NHS* proteins localized to the cellular periphery. In the developing of mammalian lens, researchers discovered continuous expression of *NHS* that became restricted to the lens epithelium in prenatal and postnatal lenses; this finding suggested that disturbances in intercellular contacts were the underlying mechanisms of cataractogenesis in NHS [19]. Additionally, the *NHS* gene appears to have multiple isoforms as a result of alternative transcription. The N-terminus of isoforms NHS-A and NHS-1A, which are implicated in the pathogenesis of NHS, have a functional WAVE homology domain that interacts with the Abi protein family, haematopoietic stem/progenitor cell protein 300 (HSPC300), Nap1 and Sra1. *NHS* knockdown resulted in the disruption of the actin cytoskeleton, and led to a striking increase in cell spreading. Conversely, ectopic over expression of *NHS* inhibited lamellipod formation. The *NHS* gene was demonstrated to be a novel regulator of actin remodeling and cell morphology, and may orchestrate actin regulatory protein function in response to signaling events during development [20]. Therefore, we speculate that X-linked cataract might represent differences in the expression of NHS.

## Conclusion

In this study, we found a novel truncation mutation in the *NHS* gene, which was predicted to be likely associated with NHS. Moreover, we reviewed the characteristics of the *NHS* gene mutations in NHS and X-linked cataract, this review illustrated that *NHS* mutations in the NHS-affected individuals typically result in premature truncation of the protein or disruption of *NHS* gene expression; and the X-linked cataract might due to the protein expression aberration. Additionally, NHS has significantly clinical heterogeneity, and the manifestations of mild NHS could be similar to those of X-linked cataract, which suggested that the combination of genome detection and the manifestations could improve the clinical diagnosis. And, the novel mutation in the *NHS* gene might highlight the understanding of the causative mutations of Nance-Horan syndrome.

## Additional files

Additional file 1: Dental dysmorphism of II: 5. A&B. Screwdriver blade-shaped incisors and mulberry-like molars are shown. C. Dental X-ray showing the overview of the tooth formation. (TIF 8647 kb)

Additional file 2: Detailed annotation of the retained 129 variants. ACMG standards were used for variant classification, and the OMIM database was used for priority analysis of the phenotype matching. (XLSX 47 kb)

Additional file 3: Mutation verification. The novel mutation was further verified in 200 control subjects by Sanger sequencing, and the *NHS* c.C4449G mutation was not detected in any individual. (DOC 1807 kb)

Additional file 4: Variants in gene *NHS* accounted for pathogenic clinical conditions. Pathogenic mutation revealed in this study and those reported in ClinVar and Cat Map databases were reviewed in the table. (XLSX 14 kb)

## Abbreviations

CADD: Combined annotation dependent depletion
Chr: Chromosome
EDTA: Ethylene diamine tetraacetic acid
ExAc: The exome aggregation consortium
GATK: Genome analysis toolkit
InDel: Insertions and deletions
NHS: Nance-Horan syndrome
*NHS*: *NHS* gene
OMIM: Online Mendelian inheritance in man
PolyPhen: Polymorphism phenotyping
SIFT: Sorting intolerant from tolerant
WES: Whole exome sequencing
